# Ageism Mediates the Relationship Between Climate and Man-Made Disasters and Loneliness

**DOI:** 10.1093/geroni/igaf122.1433

**Published:** 2025-12-31

**Authors:** Liat Ayalon, Sayani Das

**Affiliations:** Bar-Ilan University, Ramat Gan, HaMerkaz, Israel; Bar-Ilan University, Ramat Gan, HaMerkaz, Israel

## Abstract

This paper addresses two global phenomena, which pose a great challenge and threat to society at large as well as to individuals, namely natural and man-made disasters (e.g., disasters) and loneliness (defined as the subjective experience of having inadequate ties). Both the incidence and the impact of disasters and of loneliness are growing in our lives with substantial ominous prospects for the younger and older generations. Relying on data from the Longitudinal Ageing Study in India (LASI; Wave 1 (2017–18), we examine the relationship between disasters and loneliness in older persons over the age of 60. Our findings show that after controlling for demographic and environmental variables, the relationship between the two is fully mediated by ageism (e.g., stereotypes, prejudices, and discrimination towards people because of their age) and social participation (e.g., social ties and activities of social nature). Results are discussed considering theories of intergenerational relationships, stressing the need for solidarity and cooperation especially in times of threat and scarce resources.

